# How Does HDL Participate in Atherogenesis? Antioxidant Activity Versus Role in Reverse Cholesterol Transport

**DOI:** 10.3390/antiox14040430

**Published:** 2025-04-02

**Authors:** Paul N. Durrington, Bilal Bashir, Handrean Soran

**Affiliations:** 1 Faculty of Biology, Medicine and Health, Cardiovascular Research Group, University of Manchester, Manchester M13 9NT, UK; bilal.bashir2@mft.nhs.uk (B.B.); Handrean.Soran@mft.nhs.uk (H.S.); 2 Department of Diabetes, Endocrinology and Metabolism, Peter Mount Building, Manchester University NHS Foundation Trust, Manchester M13 9WL, UK

**Keywords:** antioxidant, LDL, paroxonase1, cardiovascular disease, high density lipoprotein, lipid peroxidation, atherosclerosis

## Abstract

Low-density lipoprotein (LDL) chemically modified by reactive oxygen species (ROS), for example, leaking from red blood cells in the vascular compartment, more readily crosses the vascular endothelium than does nonoxidatively modified LDL to enter tissue fluid. Oxidatively modified LDL (oxLDL) may also be created in the tissue fluid by ROS leaking from cells by design, for example, by inflammatory white cells, or simply leaking from other cells as a consequence of oxygen metabolism. As well as oxLDL, glycatively modified LDL (glycLDL) is formed in the circulation. High-density lipoprotein (HDL) appears capable of decreasing the burden of lipid peroxides formed on LDL exposed to ROS or to glucose and its metabolites. The mechanism for this that has received the most attention is the antioxidant activity of HDL, which is due in large part to the presence of paraoxonase 1 (PON1). PON1 is intimately associated with its apolipoprotein A1 component and with HDL’s lipid domains into which lipid peroxides from LDL or cell membranes can be transferred. It is frequently overlooked that for PON1 to hydrolyze lipid substrates, it is essential that it remain by virtue of its hydrophobic amino acid sequences within a lipid micellar environment, for example, during its isolation from serum or genetically modified cells in tissue culture. Otherwise, it may retain its capacity to hydrolyze water-soluble substrates, such as phenyl acetate, whilst failing to hydrolyze more lipid-soluble molecules. OxLDL and probably glycLDL, once they have crossed the arterial endothelium by receptor-mediated transcytosis, are rapidly taken up by monocytes in a process that also involves scavenger receptors, leading to subendothelial foam cell formation. These are the precursors of atheroma, inducing more monocytes to cross the endothelium into the lesion and the proliferation and migration of myocytes present in the arterial wall into the developing lesion, where they transform into foam cells and fibroblasts. The atheroma progresses to have a central extracellular lake of cholesteryl ester following necrosis and apoptosis of foam cells with an overlying fibrous cap whilst continuing to grow concentrically around the arterial wall by a process involving oxLDL and glycLDL. Within the arterial wall, additional oxLDL is generated by ROS secreted by inflammatory cells and leakage from cells generally when couplet oxygen is reduced. PON1 is important for the mechanism by which HDL opposes atherogenesis, which may provide a better avenue of inquiry in the identification of vulnerable individuals and the provision of new therapies than have emerged from the emphasis placed on its role in RCT.

## 1. Introduction

Fundamental questions about the role of lipoproteins in the pathogenesis of atheromatous diseases remain topics of active discussion. In this review, we shall focus particularly on our knowledge, or lack of it, concerning the mechanisms by which high-density lipoprotein (HDL) components by opposing oxidative modification and glycation can impede atherogenesis, firstly by preventing the passage of atherogenic lipoproteins from the circulating blood across the arterial endothelium into the subintima and secondly by decreasing the likelihood that, once there, they will cause the formation of foam cells and thence the growth of atheromatous plaques and their subsequent rupture. Because of the ubiquitous presence of HDL in tissue fluids, the antioxidative activities of HDL may also prevent the modification of other proteins, such as those of outer cell membranes, involved in the pathogenesis of many diseases other than atherosclerosis, which are the subject of other reviews and reports in this collection.

## 2. The Oxidant Theory of Atherosclerosis

The oxidant theory of atherosclerosis has recently been the subject of an excellent review from the group at La Jolla, University of California San Diego [1], where much of the concept underwent development under the leadership of the late Daniel Steinberg [2]. However, randomized clinical trials of antioxidant vitamins have yielded at best marginal benefit in the prevention of ASCVD incidents [3,4,5]. This has been widely interpreted as denying the hypothesis that oxidatively modified low-density lipoprotein (oxLDL) is a potent cause of atherogenesis. Nevertheless, whilst antioxidant vitamins can be shown to decrease the initial phase of lipid peroxidation (conjugated diene formation) on low-density lipoprotein (LDL), they may not be the best approach to prevent the later stages of LDL lipid peroxidative damage [6]. They, themselves, undergo oxidation, after which they are pro-oxidant, and they may increase potentially proatherogenic pathways, such as cholesteryl ester transfer [7].

The conversion of native LDL to oxLDL involves several stages. Antioxidants, such as vitamin E or ascorbate, prevent oxidation by themselves undergoing oxidation in preference to other substances. It was hoped that by intervening with pharmacological quantities of antioxidants of this type to prevent the earliest stage of LDL oxidation (the oxygen-free radical attack on its long-chain unsaturated fatty acyl groups of phospholipids and cholesteryl esters leading to conjugated diene formation), it would prevent atherosclerotic cardiovascular disease (ASCVD). However, they may not be the most effective or even physiological approach to prevent the subsequent generation of oxLDL. On the other hand, the hydrolysis of the breakdown products of oxygen-free radical attack, such as 9-hydroperoxy-10,12-octodecadienoic acid (9HPODE), to harmless carboxylic acids rather than allowing their breakdown into aldehydes (e.g., propanediol, hexanal, nonanal), unsaturated aldehydes (e.g., hexenal, nonenal), and their various hydroperoxy derivatives, which are damaging to the apolipoprotein B (apoB) of LDL, might be a better option. By just such a process, HDL may protect LDL from the accumulation of these breakdown products, which might otherwise adduct to the side chains of proline and arginine residues of apoB, leading to its fragmentation, following which it becomes a ligand for endothelial, macrophage, and transformed smooth muscle cell scavenger receptors [1,6]. This review will summarize some of the evidence for this supposition.

## 3. How HDL Protects Against Atherosclerosis

### 3.1. Participation in Reverse Cholesterol Transport

For many years, the inverse relationship between HDL cholesterol and ASCVD incidence has been considered to be because HDL metabolism protects against atherogenesis by being somehow rate-limiting for reverse cholesterol transport (RCT). RCT must occur because, for no known reason, the human liver secretes more cholesterol into the blood circulation than is required to fulfill the requirements of the tissues [8,9]. Therefore, if it is not to accumulate peripherally, the excess must be returned to the liver by a process that is termed RCT. It was proposed that HDL is the route by which RCT is accomplished [10]. Despite the zeal with which this idea has been pursued, evidence for a critical role for HDL in RCT has been hard to find, at least until the relatively recent finding that HDL can act as an acceptor for cholesterol exiting cells by the ATP-binding cassette transporter A1 (ABCA1 (also known as the cholesterol efflux regulatory protein)) [11,12]. Just how rate-limiting is this property of HDL in the process of RCT? Probably, the most convincing evidence that it is comes from epidemiological studies reporting that the capacity of serum to acquire cholesterol from laboratory-grown cells after removal of apoB-containing lipoproteins correlates inversely with ASCVD risk [12]. This is termed cholesterol efflux capacity (CEC). Almost certainly in the human, however, the greater part of the cholesterol acquired by HDL in this manner is rapidly esterified by lecithin: cholesterol acyltransferase (LCAT) located on HDL and then transferred to LDL by cholesteryl ester transfer protein (CETP) [13]. From LDL, any cholesteryl ester not redelivered to the peripheral tissues is returned to the liver by the LDL receptor (LDLR) and LDL-like receptor protein (LRP) [13]. Thus, LDL as well as HDL contributes substantially to RCT [14]. The balance between LDL production and catabolism, not HDL, are the major forces governing the concentration of circulating cholesterol in humans [15,16]. Evidence that the involvement of HDL in RCT is not the only nor even a major explanation for its apparent protection against ASCVD is provided by inborn errors in its metabolism [17,18,19,20,21,22,23,24,25]. In loss-of-function mutations of ABCA1 (Tangier disease), cholesteryl ester laden foam cells (macrophages) are present in many tissues, including the tonsils, spleen, and liver, accompanied by profoundly diminished HDL. A defect in HDL metabolism, so severe as to wipe out circulating HDL almost entirely, might be expected to cause remarkably premature atherosclerosis if defective RCT was critical to the process. However, atheroma is not nearly as aggressive as in heterozygous familial hypercholesterolaemia or familial dysbetalipoproteinaemia [8,9,18,19,20]. Tissues other than the arteries accumulate cholesterol earlier than the arteries. Similarly, in LCAT deficiency, despite deposition of cholesterol in the kidney often severe enough to require renal transplantation and low circulating HDL, atherosclerosis is not a prominent feature [21]. Mutations affecting specifically the apoA1 gene, whilst associated with atherosclerosis, may be because they also affect chylomicron metabolism [22,23]. Genetic CETP deficiency produces high HDL cholesterol concentrations, but any protection it affords against ASCVD is disputed [24]. The small decrease in ASCVD incidence provided by pharmacological inhibition of CETP derives more from its modest LDL-lowering action rather than from its effect in raising HDL cholesterol concentration [25]. In summary, extreme disorders of HDL metabolism, which profoundly influence its involvement in RCT, are not associated with atherosclerosis of the severity expected. It is thus reasonable to consider other mechanisms by which HDL might protect against ASCVD.

### 3.2. Impeding the Structural Modification of LDL by Oxidation and Glycation

#### 3.2.1. Source of Reactive Oxygen Species (ROS) in the Circulation

Aerobic respiration became possible as the atmosphere accumulated oxygen following the evolution of photosynthetic plants. This in turn permitted the advent of organisms with much faster metabolic rates than had hitherto been possible with simple anaerobic respiration. Oxidative metabolism, however, is not without its hazards. Until it participates in chemical reactions (oxidation), atmospheric oxygen (and thus most dissolved oxygen) exists as a couplet in which two oxygen molecules, each having six electrons in their outermost shell, share with each other two of these electrons to completely fill their outermost shells, creating stability. When oxygen (O_2_) combines by sharing electrons with other atoms, the first stage must inevitably be the creation of superoxide in which the couplet has gained a single electron, but has yet to acquire an additional electron to complete its outer shell. It is thus highly reactive, having at that stage a similar outer shell to fluorine and thus a similar predilection for combining with carbon at alkene bonds, such as those in linoleate and arachidonate [18]. A typical human consumes nearly 400 L of oxygen every day, a substantial proportion of which enters the blood circulation, where it combines reversibly with hemoglobin in erythrocytes. For this to occur, the iron in hemoglobin must remain in the Fe^ll^ state (ferrous). If only a small proportion of Fe^ll^ is oxidized irreversibly to Fe^lll^ (ferric) (methemoglobin), significant quantities of superoxide can be generated (as follows) with the potential to damage other vital molecules [26,27].

**Hb-Fe^ll^ + O_2_ → Hb-Fe^lll^ + O_2_^•−^**

Superoxide rapidly combines with hydrogen ions to form hydrogen peroxide.

**2 O_2_^•−^ + 2H^+^ → H_2_O_2_ + O_2_**

Technically, hydrogen peroxide is not an oxygen-free radical, but it is water soluble and highly reactive. The term reactive oxygen species (ROS) is used to encompass true free radicals, such as superoxide, and their often more reactive and ubiquitous derivatives, such as hydrogen peroxide [28]. To overcome damaging ROS, the red cell contains an active pentose phosphate pathway (PPP) producing the reducing agent, NADPH, which maintains glutathione in a reduced state to neutralize superoxide [29]. Nevertheless, some must inevitably escape glutathione to react with protons to become hydrogen peroxide, which can readily diffuse from red cells to react, for example, with the alkene groups in phosphatidyl choline and cholesteryl esters of LDL to generate oxidatively modified LDL (oxLDL). HDL may provide an alternative pathway for the harmless removal of these lipid peroxides escaping across the erythrocyte outer cell membrane [30]. The PPP itself, despite its generally protective role, may also contribute to the chemical modification of proteins, such as apoB, because it produces metabolites linked to glycation (see later).

#### 3.2.2. Oxidative Modification of LDL in the Circulation

**Figure 1:**
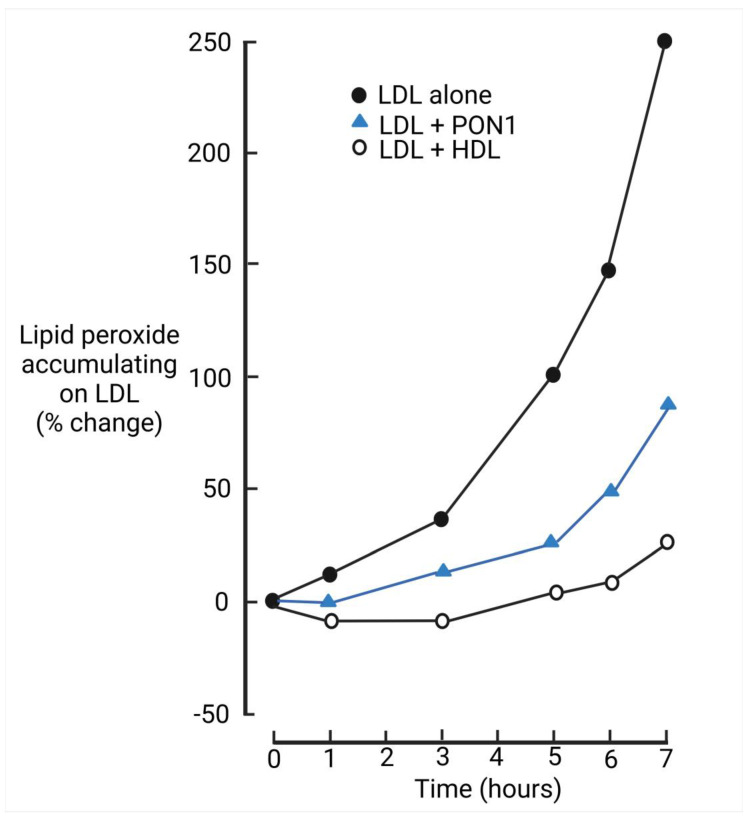
The generation of lipid peroxides over time when LDL is incubated with CuSo_4_ 5 μM alone, with HDL and in the presence of paraoxonase 1 (PON1). Data from reference [31].

There appears to be no disagreement about the notion that HDL ameliorates the biological effects of LDL oxidation that was observed in the earliest reports that LDL could damage cells in tissue culture [1]. Figure 1 shows an experiment where the oxidation of LDL by Cu^II^ ions has been prevented when HDL is also present [31]. OxLDL was detected by the method of el-Saadani, which makes use of the color reagent of a commercially available test kit for cholesterol [32]. This method measures the accumulation of the products of lipid peroxidation. In the presence of HDL, these largely disappear. They are fatty acyl hydroperoxides typically derived from linoleate and arachidonate, which rapidly break down to form ketones and aldehydes capable of adducting to the side chains of proline and arginine residues of apoB, leading to fragmentation of the apoB molecule, which thereby becomes a ligand for scavenger receptors, such as scavenger receptor class B type 1 (SR-B1 aka SCARB1; lectin-like oxidized LDL receptor1, LOX-1) [1]. The earlier phase of lipid peroxidation, namely conjugated diene formation detectable by ultraviolet (UV) absorbance, which is delayed by oral administration of antioxidant vitamins [6,7,33], is unaffected by HDL, whereas it is by oral administration of antioxidant vitamins [6,7,33]. This effect of antioxidant vitamins on conjugated diene formation is, however, short-lived compared to that of HDL on the later phases of lipid peroxide breakdown.

Probably, in the presence of HDL, phospholipid hydroperoxides are hydrolyzed to carbonic acids, and thus ketones and aldehydes do not accumulate [34,35,36]. Whatever the mechanism by which oxidative modification of LDL is impeded when HDL is present, it is likely to require transfer of phospholipid hydroperoxides from LDL to HDL presumably by phospholipid transfer protein and/or CETP [37]. This is extremely important, because, were a peroxidized phospholipid, for example, phosphatidylcholine (lecithin), to remain on LDL, its hydrolysis by phospholipase A2 (aka platelet activating factor acetylhydrolase) present on LDL would release the peroxidized fatty acyl group at Sn2 to breakdown into aldehydes and ketones on LDL and moreover leave behind lysophosphatidylcholine (lysolecithin) which is itself intensely damaging to LDL and tissues [38]. HDL is, however, also known to possess phospholipase A2 activity [39]. Thus, what can be the advantage of transferring phospholipid hydroperoxides to it from LDL? One important answer is that HDL, with its intensely hydrophobic environment maintained by the strongly detergent apolipoprotein A1 (apoA1) provides a safe environment for the release of toxic lipids, such as lysophatidylcholine. This must be the case because cholesterol secreted by the liver, mostly in VLDL, is free (unesterified), yet most of that in the circulation is esterified [8]. This is accomplished by LCAT present on HDL in the following reaction:

Cholesterol + Phosphatidylcholine → Cholesteryl ester + Lysophosphatidylcholine.

LCAT on HDL also esterifies free cholesterol effluxing from peripheral cells, which is initially taken up by small HDL particles (preβ-HDL) where it is esterified so that it can be packed into the intensely hydrophobic core of these particles to allow more free cholesterol to join their surface layer [40,41]. Thus, the particles enlarge to become HDL_3_. Substantial gram-range quantities of cholesterol are esterified on HDL in this way every day and thus the stoichemical production of potentially toxic lysophosphatidylcholine is similarly great [8]. The production of lysophosphatidyl choline on HDL by LCAT, is a physiological process, which presumably does no damage to tissues. Thus, HDL must be a safe environment in which to hold lysophosphatidylcholine until it can be released to hepatocytes during the passage of HDL through the liver. Additionally, there is no apoB located on HDL to damage. Interestingly, much of the phospholipase A2 activity on HDL often attributed to the enzyme, phospholipase A2, may be due to paraoxonase 1 (PON1) [39]. Partially purified PON1 maintained in a lipid environment was reported by us to be highly effective in impeding Cu-induced oxidation of LDL lipids (Figure 1) (see [6] for discussion). LCAT, apoA1, and a hydrophobic, lipid environment are likely to be essential for the hydrolytic activity of PON1 towards lipid peroxides.

The composition of HDL varies greatly both within and between individuals. Proteomic investigations have revealed at least a hundred potential HDL components. Its antioxidant activity reflects the balance between PON1 activity, LCAT, and apoAI on the one hand and on the other the presence of pro-oxidant myeloperoxidase and of serum amyloid A (SAA) and apoAII, both of which displace PON1 from HDL [6]. HDL, deficient in PON1, rich in both SAA and myeloperoxidase with a low ratio of apoAI to AII, has been called proinflammatory HDL because of its association with acute and chronic inflammatory states, such as infection, rheumatoid arthritis, and autoimmune diseases. This type of HDL may also have a limited capacity to impede LDL oxidation and glycation or to promote cholesterol efflux, say from macrophage foam cells (see later).

#### 3.2.3. Oxidative Modification of LDL in the Tissues

Cells in tissue culture oxidatively modify LDL [1,42,43]. In the case of endothelial and smooth muscle cells in tissue culture, this may simply be due to leakage of ROS from their cytoplasm. On the assumption that ROS also escape from cells in vivo, this would account for the generation of oxidatively modified LDL in the tissue fluid. Added to this, neutrophils and macrophages actively produce ROS to shower on invading pathogens and probably macromolecules recognized as misplaced or effete as a component of the host defense mechanism [44].

#### 3.2.4. Lipoprotein (a)

Although there is a positive association between ASCVD incidence and lipoprotein(a) (Lp(a)), the mechanism remains elusive. Oxidized phospholipids have been reported to accumulate preferentially on Lp(a) in comparison to other LDL subclasses (apoB-containing lipoproteins) [1]. The rate at which LDL isolated from different individuals accumulates lipid peroxides in the presence of Cu^II^ differs greatly [33]. One factor that may contribute to this variation, which has never been explored, is the proportion of Lp(a) present in LDL used in these experiments. Lp(a), once it has crossed the arterial endothelial barrier, is retained there longer than more buoyant LDL [45], which may increase the extent to which it undergoes lipid peroxidation there.

#### 3.2.5. Small, Dense LDL

In terms of hydrated density, small dense LDL (SD-LDL) is similar to Lp(a). Additionally, in common with Lp(a), SD-LDL is not cleared by the LDL receptor, spending more time in the blood and lymphatic circulation than more buoyant LDL [46]. SD-LDL is more susceptible to oxidative modification [47] and to glycation [48,49,50], which may be two sides of the same coin (see later). Circulating SD-LDL is increased in parallel with more buoyant LDL concentration in, for example, familial hypercholesterolemia, and as a proportion of LDL as a whole is increased in metabolic syndrome and diabetes. It contributes to serum apoB100 concentration, but not greatly to LDL cholesterol. Unless a method for measuring SD-LDL is available, its presence in increased concentrations can generally be expected when triglycerides are increased (perhaps only modestly) and HDL cholesterol is low [46,51,52].

#### 3.2.6. LDL Glycation

In health approximately 3–4% of plasma LDL is glycated when the concentration is determined by boron affinity chromatography and apoB immunoassay [53]. The concentration is thus normally approximately 3 mg/dL. The percentage of serum LDL, which is glycated and its concentration rise as glycated hemoglobin increases; both are thus raised in diabetes [53]. In untreated familial hypercholesterolemia, whilst the percentage of LDL that is glycated, is relatively normal, the concentration of glycated LDL (glycLDL) is frequently as high as in diabetes because of the raised LDL levels [53]. Glycated LDL can cause foam cell formation and binds to the same receptors as oxidatively modified LDL (oxLDL) [54]. It may thus have similar atherogenicity. Allowing for methodological differences and definitions of how irreversibly glucose is bound to LDL, glycLDL concentration in serum is similar to or may even exceed that of oxLDL. It is thus likely to be a major player in atherogenesis and one that has been neglected. Just as SD-LDL undergoes oxidative modification more readily than more buoyant LDL, SD-LDL is more susceptible to glycation [48,49,50]. Moreover, HDL ex vivo has been reported to protect LDL against glycation [55]. Furthermore, HDL from people in the upper half of the PON1 activity distribution impedes glycation more effectively than HDL from the lower half of its population distribution [55].

Glycation is itself a process that generates ROS [56,57]. Thus, even when incubation of LDL with glucose occurs under nitrogen, not only glycLDL, but also oxLDL may be produced [58,59]. The explanation for this might be mechanistically similar to the effect of PON1 in decreasing LDL oxidative modification, because during glycation of LDL by glucose and its metabolites, the same nucleophilic side chains of lysine and arginine in apoB are attacked as are by aldehydes and ketones generated by lipid peroxidation. Both the generation of oxLDL and glycLDL may be accelerated when both ROS and glucose and its metabolites are present [59,60,61,62,63,64,65,66,67,68].

Despite employing higher glucose concentrations than are likely to occur in vivo, glycation of LDL ex vivo in the absence of oxygen is a slow process [48,49,50,51,52,53,54,55]: too slow to account for the proportion of LDL that is glycated in serum and that must be formed in the few days between hepatic VLDL secretion, conversion to LDL, and its catabolism [8]. Ex vivo, LDL glycation experiments have generally been performed under nitrogen in the absence of atmospheric oxygen, and thus ROS may not be present to the same extent as in vivo. In the presence of Cu^II^, glycation of LDL is accelerated [58]. Glycation may also proceed more rapidly in vivo because more reactive metabolites of glucose are present. These can accelerate glycation of LDL. Interestingly, the erythrocyte PPP and glycolytic pathways are likely to be significant sources of ROS in the circulation. Gluconolactone has been shown to rapidly glycate proteins [60], and phosphogluconolactone generation is the first step in the PPP [61]. In this context, the lactonase activity of PON1 should not be overlooked in contemplating the mechanism by which HDL protects LDL against glycation. Methylglyoxal another potent glycating agent, is produced in the glycolysis pathway by nonenzymatic phosphate removal from two of its intermediates, glyceraldehyde 3-phosphate and dihydroxyacetone phosphate [62,63,64,65,66,67,68]. It is also a product of the PPP. Both the PPP and glycolysis are located in the erythrocyte cytoplasm, increasing the likelihood of the escape of their intermediates across the outer cell membrane of red blood cells coming into contact with LDL in the circulation [69]. They might explain the high proportion of glycLDL circulating even in nondiabetic people, despite the slow rate of LDL glycation in experiments with glucose itself. Such experiments, however, suggest that HDL, particularly PON1—rich HDL, is likely to impede apoB glycation [55].

### 3.3. Mechanism by Which the Antioxidative Action of HDL Protects Against Atherogenesis

#### 3.3.1. Impeding the Passage of Atherogenic Lipoproteins Across the Arterial Endothelium

**Figure 2:**
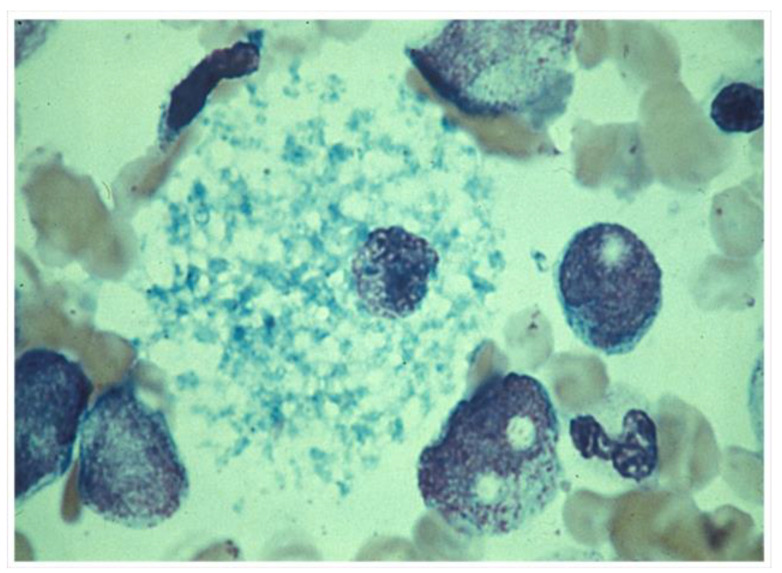
Foam cell in bone marrow aspirate from a patient with severe hypertriglyceridemia [70].

The arterial endothelium is not fenestrated, unlike endothelia in the reticuloendothelial system, which certainly do allow the passage of lipoproteins up to chylomicron dimensions. When chylomicrons circulate in excess, the smaller ones not only cross the fenestrated endothelia of liver, spleen, and bone marrow, but can be taken up by macrophages there, leading to foam cell formation and hepatosplenomegaly due to engorgement with lipid. Figure 2 shows a macrophage foam cell in the bone marrow in familial lipoprotein lipase deficiency [70].

**Figure 3:**
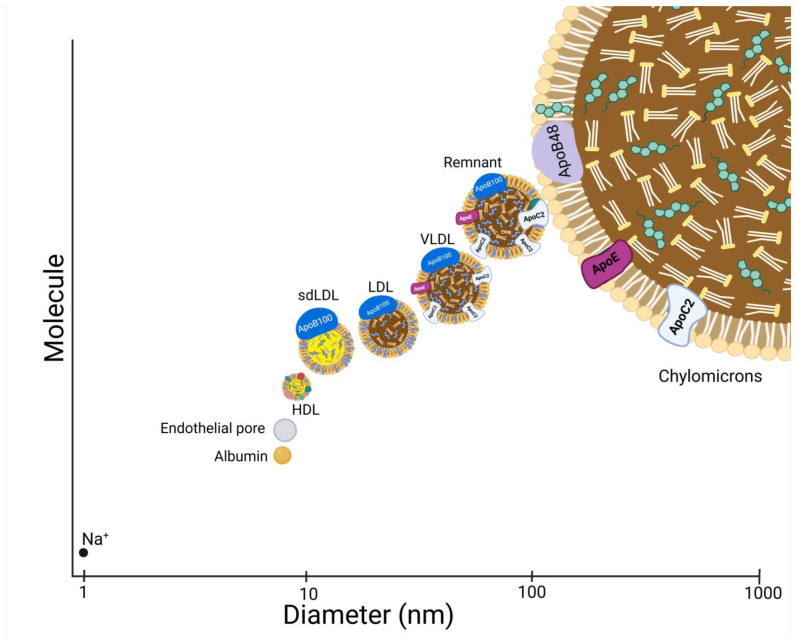
The molecular dimensions of lipoproteins, albumin, and sodium. The pore size of arterial endothelium was considered to be <12 nm, but the existence of anatomical pores in healthy, intact, noninflamed arterial (as opposed to capillary) endothelium now seems unlikely. HDL and LDL (particularly oxLDL and glycLDL) cross by receptor-mediated transcytosis. See text for references.

For many years, lipoproteins and other macromolecules were believed to cross the vascular endothelium by a simple process of filtration. It was reported that lipoproteins with a diameter exceeding 12 nm were excluded from crossing intact, healthy vascular endothelial membranes (Figure 3) [71]. This explained why HDL, with its molecular diameter of 7–13 nm and components of HDL, such as apoA1, are detectable in tissue fluid and lymph at concentrations at approximately one-fifth those in plasma [72]. Some other components, such as PON 1, are decreased proportionately more than apoA1, because of sequestration by cell membranes [73]. However, LDL, theoretically too large to cross intact vascular endothelia, is also present in tissue fluid, albeit at only about one tenth of its plasma concentration [72,74]. Indeed, if LDL is to fulfill its role in supplying cholesterol to tissues, its presence in tissue fluid is essential. Furthermore, the cholesterol present in atheromatous plaques is derived from LDL cholesterol [1,2]. Both apoB100 and apoB48 have been identified in atheromatous lesions, suggesting that not only LDL, but also small remnants of gut-derived lipoproteins can participate in atherogenesis [75,76,77]. Chylomicrons (Sf > 400) have a diameter of 100–10^5^ nm. Chylomicron remnants, which in health are briefly present in the blood circulation as a small component of VLDL (Sf20–400), vary in diameter from 30–90 nm. To explain the presence of lipoproteins larger than HDL in tissue fluid, it was postulated that damage to the endothelial junctions or loss of endothelial cells was required to permit LDL and even larger lipoproteins to cross [78,79]. Such a situation might occur in established (mature) lesions, but is unsatisfactory to account for LDL as the cause of the precursor fatty streak and earlier atheromatous lesions occurring when the endothelium is intact. The theory that the passage of LDL across the arterial endothelium, leading to atherogenesis, is the result of mechanical or chemical damage to the endothelial junctions or loss of endothelial cells, particularly occurring at sites in the arterial tree where atherosclerosis frequently occurs, whilst undoubtedly true in established atheroma [78,79,80], is unlikely to explain initial lesion formation when the endothelium is intact. Another theory proposes that inflammation is the initiating event, increasing endothelial permeability to permit the passage of monocytes and lipoproteins across the arterial endothelium [81,82,83]. Many authors have written about endothelial dysfunction defined as a deficiency of nitric oxide (NO) [84,85]. NO bioavailability is decreased when superoxide or hydrogen peroxide reacts with it to form peroxynitrous acid (ONOOH), toxic to cellular structures.

**O_2_• + NO → ONOO**^−^

**2 H_2_O_2_ + NO → ONOO**^−^** + H_2_O**

Decreased NO bioavailability will undoubtedly affect the inflammatory responses of the arterial wall, particularly at sites prone to the development of atherosclerosis [84,85]. The essential question, however, is at what stage in the disease process? There is a vast literature on the inflammatory cytokine responses that occur during the development of atheromatous lesions [83]. Leucocyte chemoattractant chemokines followed by interleukin-1β (IL-1β) produced by monocytes recruited from the blood circulation and tissue macrophages within the lesion are heavily implicated [83]. The simple fact remains, however, that entry of LDL into tissues physiologically cannot depend on inflammation. LDL can cross the arterial endothelium without the need for inflammation; indeed, it has to do so if it is to perform its physiological function of delivering cholesterol to the tissues. Furthermore, there is no animal model of atherosclerosis in which the inflammatory response or individual inflammatory cytokines can be tested as a cause that does not involve an animal that has been rendered hypercholesterolemic [86]. As Anitschkov wrote, ‘there can be no atheroma without cholesterol’ [87].

Certainly, in the early stages of atherogenesis, there must be another explanation for how LDL crosses the arterial endothelium besides increased porosity due to damage or as a response to inflammation. The earliest lesion in atherosclerosis is the fatty streak, and by this stage, LDL has already crossed the arterial endothelium in quantities sufficient to produce cholesterol deposits. The location of these subendothelial deposits at junctions, at sites where arteries are tethered and not cushioned, and the very fact that the streaks are parallel with the direction of blood flow suggests that hemodynamics plays some part in their pathogenesis [88,89,90]. However, fatty streaks are uncommon in young people in populations in which cholesterol levels are low and where later atherosclerotic complications are few [91,92]. This makes it highly likely that LDL, (in particular, oxLDL—see later) initiates fatty streaks by penetrating or at least interacting with the arterial intima before it expresses adhesion molecules and secretes chemokines, leading to the recruitment of circulating leukocytes [93]. When monocytes-macrophages subsequently, of course, do infiltrate atherosclerotic plaques, they take up oxLDL and form “foam cells” that in turn release inflammatory mediators (tumor necrosis factor, interleukin-6, soluble intercellular adhesion molecule 1 and circulating vascular cell adhesion molecule 1), reinforcing the process. Nonetheless, the importance of oxLDL penetrating intact arterial endothelium is that it is likely to initiate the whole process. Furthermore, the importance of oxLDL from the circulation crossing the arterial endothelium is not confined to fatty streak generation in the initial stage of atherogenesis. It may also be essential for the mature atheroma to grow concentrically along the arterial wall. Inflammatory (monocyte recruitment and subsequent foam cell formation), maladaptive wound-healing responses (smooth muscle cell recruitment and subsequent fibrosis), and antibodies to oxLDL [1,81,83] are clearly essential, but the growth of the lesion occurs at its edges (shoulders on 2-dimensional microscopy) by a similar mechanism to the initiation of fatty streaks; the passage of oxLDL across relatively normally functioning arterial endothelium.

Thus, key to the initiation and expansion of atheroma is the subintimal influx and accumulation of LDL leading to the recruitment of blood monocytes. Both are encouraged in the presence of hyperlipidemia when the quantity of intimal LDL and the oxidative potential of the intima exceed the capacity of macrophages to remove it.

As undoubtedly HDL and LDL constitute a system for delivery and removal of cholesterol to and from tissues, they must be capable of transport across vascular endothelia into tissue fluid, and clearly this can no longer be considered to rely on simple filtration across the vascular endothelium. Furthermore, much of what has been written about the filtration of macromolecules relates to their passage across capillary endothelia, but there is greater resistance to the passive transfer of macromolecules across the arterial endothelium where atherogenesis occurs. Transfer is therefore now believed to occur by transcytosis and pinocytosis [94,95,96,97,98,99,100,101,102,103,104,105,106,107,108]. In transcytosis, molecules bind to receptors on, the apical surface of endothelial cells and are transported in endosomes through the cytoplasm to be released at their basal surface. Receptors proposed for transcytosis include the LDL receptor [94,95]. Because this receptor binds both apoB100 and apoE [9], both LDL (via apoB100) and chylomicron remnants (via apoE) could cross by this mechanism. However, the importance of this route for atherogenesis is questionable, firstly because it is saturable and thus only likely to operate when LDL cholesterol levels are lower than in adults. At typical adult concentrations of serum LDL cholesterol, the LDL receptor will be downregulated. Furthermore, in familial hypercholesterolemia, LDL receptor expression is impaired, whilst atherogenesis is rife [9].

**Figure 4:**
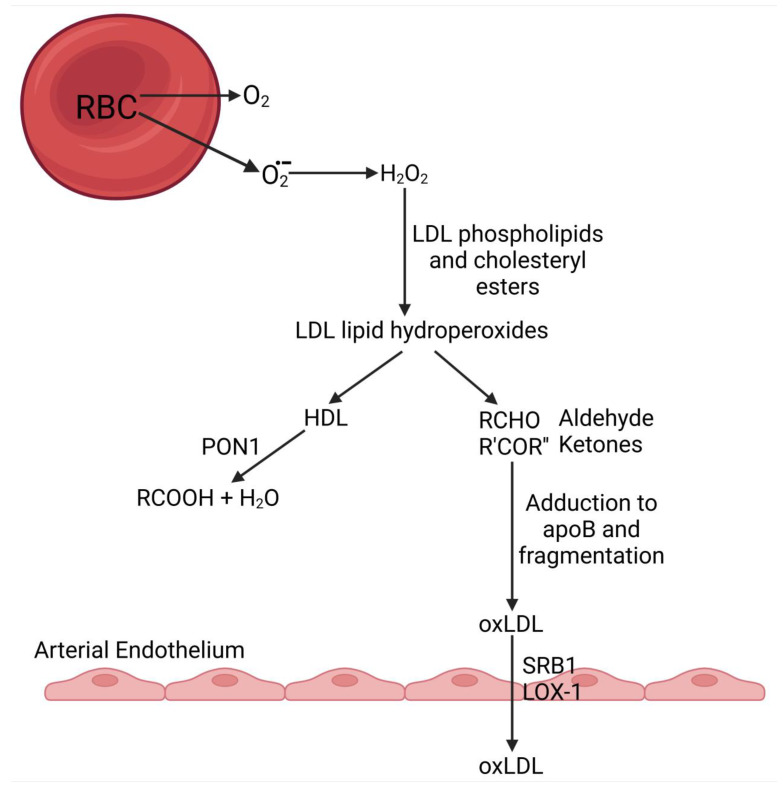
Events leading to the passage of LDL across the arterial endothelium. ROS are released into the circulation, for example, from red blood cells. There they react with the unsaturated acyl groups of lipoprotein phospholipids and cholesteryl esters on LDL to form lipid peroxides. The apolipoprotein B of LDL adducts to the ketones and aldehydes produced by the breakdown of lipid peroxides and then undergoes fragmentation. The resultant oxidatively modified LDL (oxLDL) undergoes endothelial transcytosis as the result of binding to scavenger receptors (SR-B1 and LOX-1). HDL can protect LDL against oxLDL formation.

Another receptor-mediated route proposed for endothelial transcytosis, proposed soon after the LDL receptor fell out of favor to serve this purpose, is the scavenger receptor class B1 (SR-B1 aka SCARB1) (Figure 4) [94,95,96,97,98,99,100,101,102]. This potentially allows the passage of both LDL and HDL into the arterial subintima. Mice overexpressing endothelial SRB1 (as opposed to hepatic or macrophage SR-B1) were protected against atherosclerosis. The SR-B1 is not saturable, and thus it permits LDL and HDL to cross the arterial endothelium to an increasingly greater extent as their serum levels increase. Passage of LDL across the arterial endothelium can also be mediated by direct pinocytosis via caveolae [102], by the actin receptor-like kinase 1 (ALK1), and by LOX-1 [102,103,104,105]. Of all these mechanisms, LOX-1 has recently been proposed to be responsible for the largest proportion of the LDL that crosses the arterial endothelium [102,103,104,105]. OxLDL is the preferred ligand for both SR-B1 and LOX-1, and its passage through the arterial endothelium by these routes is critical to the initiation of atheroma. GlycLDL also crosses from the arterial lumen by transcytosis [106,107], thus contributing to atherogenesis. HDL is transcytosed across the arterial endothelium not only by the SR-B1 receptor, but also by the ATP-binding cassette transporters A1 and G1 (ABCA1 and ABCG1) [94,98].

All these receptors can permit the passage of LDL and HDL across the arterial endothelium in either direction and could thus mediate the return of LDL or HDL cholesterol to the circulation. Whether transcytosis of LDL adequately explains why atheroma develops under intact, uninjured endothelium has been questioned [108]. It is argued that the source of the LDL cholesterol may be from the blood vessels (vasa vasorum) in the artery wall rather than the arterial lumen. Atherogenesis may thus occur when this cholesterol accumulates because its clearance rate by transcytosis into the arterial lumen lags behind its rate of arrival from vasa vasorum.

Does transcytosis explain why there is more HDL than LDL in tissue fluid? Size must play some part. Presumably, the smaller HDL particles undergo pinocytosis and receptor-mediated endocytosis more readily than the larger LDL. Whether lipoproteins larger than LDL can cross the intact arterial endothelium is unresolved, but is probably unlikely, except perhaps in the case of smaller VLDL and chylomicron remnant particles. More certain is that the relation between triglyceride-rich lipoproteins and atherosclerosis is due to the generation of cholesterol-depleted SD-LDL, which goes unobserved in epidemiological studies linking triglyceride levels to ASCVD incidence. SD-LDL is generated by CETP and the action of lipases during the circulation of triglyceride-rich lipoproteins [46,51]. Critical for atherogenesis is likely to be that the receptors involved in endothelial transcytosis, apart from the LDL receptor, which is generally down-regulated (see back), favor ox LDL and glyc LDL over native LDL [94,95,96,97,98,99,100,101,102,103,104,105,106,107,108].

Several studies have reported that transcytosis of LDL mediated by mechanisms other than the LDL receptor is impeded by HDL. This was first observed in fibroblasts [109], but applies also to arterial endothelial cells [110]. The mechanism is highly likely to involve the prevention of LDL oxidation by HDL, reducing the likelihood of LDL binding to SR-B1 and other receptors, showing a preference for binding to oxLDL over native LDL [94,95,96,97,98,99,100,101,102,103,104,105,106,107,108]. It represents a potential mechanism preventing atherogenesis. Just as the role of HDL in encouraging the efflux of cholesterol from cells is enhanced by HDL with higher PON1 content (see next section), so also may be the flux of ox LDL and glyc LDL across the arterial endothelium into the subintima. The inhibition by HDL and purified PON1 of the ox LDL-induced transmigration of monocytes across aortic endothelial cell monolayers was first demonstrated in cocultures by workers from Los Angeles [36].

#### 3.3.2. Impeding Foam Cell Formation in the Arterial Wall

Foam cells are present throughout the atheromatous process from its origin in fatty streaks to the mature lesion with its fibrous cap overlying a lake of cholesterol [78,79,111,112]. Foam cells appear to orchestrate through the elaboration of cytokines and chemokines much of the growth of the plaque, attracting into it the monocytes and undifferentiated smooth muscle cells, both of which transform themselves into foam cells, swelling their numbers in the developing lesion. Foam cells are phagocytic macrophage-type cells that have within their cytoplasm abundant droplets of cholesteryl ester. As their numbers increase, they undergo apoptosis and necrosis to form the extracellular deposits of cholesteryl ester within the atheroma. Growth of the lesion is believed to take place at its edges where foam cell formation is most active, allowing the plaque to extend itself around the circumference of the artery very much as the original fatty streak had progressed. Smooth muscle cells entering the lesion not only swell the ranks of the foam cells, but also themselves transform into fibroblasts. Fibrosis, which may represent an aberrant wound-healing response, leads to the development of the fibrous cap overlying the lesion. However, the coup de grace, the rupture of the fibrous cap, leading to thrombosis and acute clinical events, is frequently in its shoulder region, where foam cells are abundant and may be mediated by metalloproteinases secreted by these foam cells, weakening fibrous tissue in that region.

**Figure 5:**
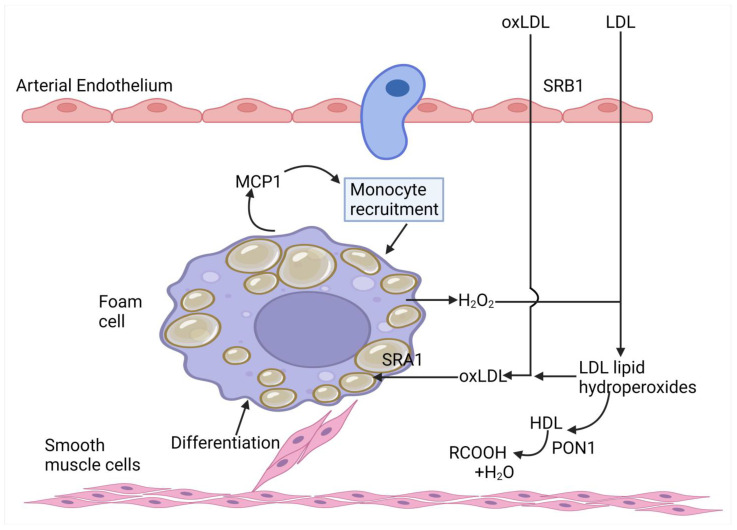
OxLDL arriving beneath the arterial wall attracts monocytes to enter from the blood circulation. These transform into monocyte-macrophages. More oxLDL is created by the generation of ROS by these monocyte-macrophages, which take up oxLDL via scavenger receptors (CD36 and SR-A1) to become foam cells. These permit the growth of the atheromatous lesion at its edges, whilst centrally the chemokines and cytokines they secrete attract increasing numbers of monocytes and other inflammatory cells into the lesion, stimulate smooth muscle proliferation, and transform into either fibroblasts secreting collagen or more foam cells. Foam cells eventually undergo necrosis or apoptosis, leaving behind the central lake of cholesteryl ester with its overlying fibrous cap.

Our understanding of how foam cells form has undergone a similar evolution to that of how receptors were recognized to be necessary for the transcytosis of LDL by arterial endothelial cells. In the case of monocyte-macrophages, it was found that the uptake of LDL by the LDL receptor was too slow to cause foam cell formation. Chemically modified LDL was, however, rapidly taken up, leading to foam cell generation (Figure 5). Naturally occurring chemical modifications of LDL that cause foam cell formation are oxLDL, glycLDL, and malondialdehyde-modified LDL. These are ligands for cluster differentiation 36 (CD36 aka scavenger receptor class B Type 3 (SCARB3)), SRA-1 and LOX-1 [111,112].

Because rapid uptake of LDL by monocyte-macrophages depends on the chemical modification of LDL, it might be expected that limiting the chemical modification of LDL would reduce the likelihood of its uptake, and both HDL and PON1 have been reported to diminish LDL-induced foam cell formation [2,36]. PON1-rich HDL can decrease the generation of both oxLDL and glycLDL [34,36,55].

The formation of foam cells, whether from monocyte-macrophages or smooth muscle cells, represents the balance between LDL uptake and the egress of cholesterol from the cells [113]. As discussed earlier, this cholesterol efflux may be through ABCA1 to small HDL and even smaller prebeta HDL particles, where it is packed into a central core after undergoing esterification by LCAT. It can also be accomplished by macrophage secretion of apolipoprotein E (apoE) [114]. HDL and paraoxonase have been reported to increase the efflux of cholesterol from macrophages [115]. The authors of the report concluded that the generation of lysophosphatidyl choline by paraoxonase, together with its effect in protecting against peroxidation of HDL and cellular membranes, increased the probability of HDL binding to and receiving cholesterol effluxing from macrophages. PON1 is known to redistribute from HDL to cell membranes [116]. As with arterial endothelial uptake of oxLDL, physical forces may contribute to the likelihood of foam cell formation within the arterial wall. Evidence suggests that macrophage cell types cultured under pressure take up oxLDL more readily to become foam cells [117].

### 3.4. Antioxidant Components of HDL

From the foregoing, HDL, by virtue of its capacity to hydrolyze lipid peroxides to harmless carboxylic acids before their breakdown into aldehydes and ketones, can decrease the formation of oxLDL both in the circulation and within the arterial wall. Foremost amongst the components of HDL likely to be important for the protection of LDL against oxidative modification appears to be PON1 [6,118]. Essential for its activity against hydrophobic substrates is the hydrophobic environment provided by the powerful detergent activity of apoA1 and by the enzyme LCAT, which creates the hydrophobic core of HDL.

**Figure 6:**
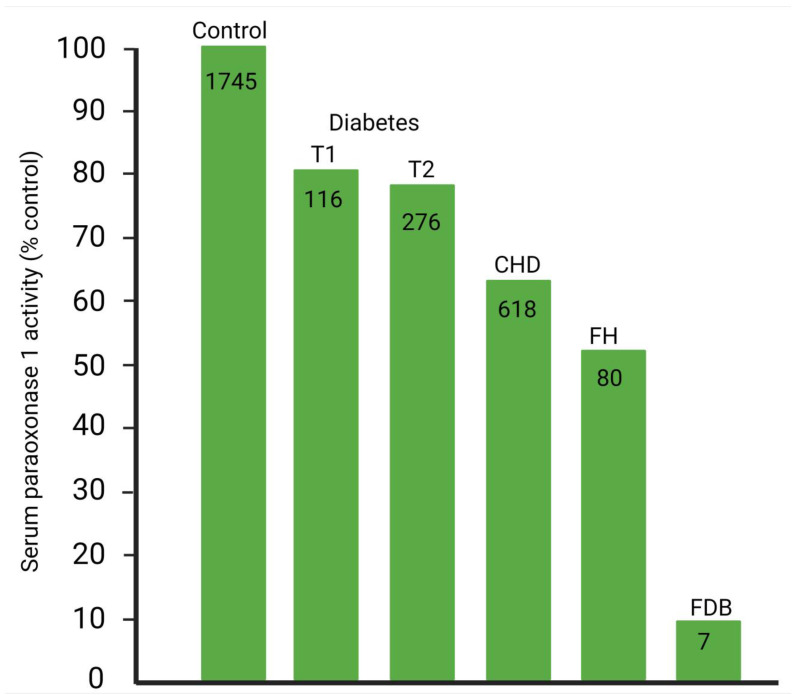
Median serum PON1 activity measured in our laboratory expressed as a percentage of control in Type 1 and Type 2 diabetes, coronary heart disease, familial hypercholesterolemia (FH), and familial dysbetalipoproteinemia (FDB). The number of individuals studied is shown. The controls were men recruited from the general population who did not experience acute myocardial infarction during follow-up. In 555 controls who were considered healthy at the time of venipuncture, median serum PON1 activity was higher at 184.4 U/L. Data from references [50,127,128,129,130,131,132,133].

PON1 was discovered in serum in 1953 and originally classified as ‘A’ esterase to distinguish it from esterases that were blocked by organophosphates, such as paraoxon (then used extensively in agriculture as a pesticide as its precursor, parathion) [119]. There followed extensive studies of its role in toxicology, which revealed the wide range of organic substrates it could hydrolyze and its location on HDL [6,120,121,122,123,124]. PON1 was found to be increasingly present in atheromatous lesions as they progressed [125]. PON1 activity is decreased in coronary heart disease [126,127,128,129], familial hypercholesterolemia [130], type1 and 2 diabetes mellitus [131,132], familial dysbetalipoproteinemia [133] (Figure 6), metabolic syndrome [134,135,136,137,138,139,140], polycystic ovary syndrome [141], and chronic renal disease [142]. PON1 is one of a family of paraoxonases, the other members of which are PON2, an intracellular enzyme, and PON3, a minor component of HDL. In 1991, PON1 partially purified from HDL was reported to prevent the accumulation of lipid peroxides on LDL under oxidizing conditions [34]. It was subsequently found that PON1 prevented minimally oxidized LDL-induced migration of human blood monocytes through a layer of cultured endothelial cells and decreased oxidation products of phosphatidyl choline [36].

PON1 has several polymorphisms, of which one has been frequently shown to be associated with ASCVD. This occurs as the result of whether glycine(Q) or arginine (R) is present in position 192 of its amino acid sequence. This polymorphism is also designated rs662. The 192R isoenzyme of PON1 is less active in protecting LDL against oxidative modification [6,143] and is more closely associated with ASCVD [6,144,145]. However, the 192Q enzyme confers protection too. It is thus the combined serum concentration of both isoenzymes, which in epidemiological studies is at least as predictive of ASCVD incidence as HDL cholesterol, particularly in diabetes and established ASCVD [6,146]. Activity assays that employ substrates to which PON1 does not display substrate specificity have high activity, and are nontoxic, such as phenyl acetate, have the most clinical utility [6,147]. One extreme example of nondiscordance between HDL cholesterol and PON1 activity is Tangier disease (see back), where serum HDL cholesterol is almost entirely absent, but PON1 activity is present within the normal range [148].

PON1 not only displays esterase activity, but has even greater lactonase activity and may have its evolutionary origin as a lactonase [149]. Its antiatherosclerotic role could thus be as an antioxidative enzyme, as a lactonase or both [118].

## 4. PON1 and ASCVD Causality

Epidemiological studies report that ASCVD cases have lower PON1 activity measured with paraoxon or phenyl acetate as a substrate compared to controls [6]. Activity of PON1 has generally been found to be a better discriminator than concentration. Most important amongst these are the prospective studies revealing that the association between lower PON1 activity and ASCVD is not the consequence of the ASCVD event or its treatment or due to lower levels being associated with survival, but that it predates the clinical event [6,128,129,146,150,151]. Furthermore, low PON1 activity may explain cases of ASCVD when HDL cholesterol is high, which were previously thought anomalous [152]. However, epidemiology cannot prove causation. In theory, Mendelian randomization studies come closer to revealing causation, and the Q192R gene variant presents an opportunity for this type of investigation. There have been more than 100 reports, which have, with considerable consistency, demonstrated an association between the 192R (which is less effective than 192Q in decreasing LDL oxidation) and the likelihood of ASCVD [144,145]. However, 192R (even though less active than Q in hydrolyzing lipid peroxides) does still protects against oxidative modification of LDL, albeit less effectively than 192Q. Thus, higher concentrations can overcome its potentially adverse genotype effect [128]. Thus, the effect is smaller than might be expected from in vitro experiments in which similar concentrations were compared. Many other factors, including dyslipidemias and diabetes, are involved in determining PON1 activity, and these may be more influential than any gene so far discovered that modifies PON1 activity or concentration [153,154].

Animal experiments provide the strongest evidence that the epidemiologically observed inverse relationship between serum PON1 concentration and ASCVD is causal.

1. Serum PON1 activity varies greatly throughout the animal kingdom. Birds lack serum paraoxonase activity, whereas humans have substantial amounts, and rabbits, for example, even more. Human HDL protects LDL against oxidative modification, avian HDL fails to do so [155].
2. PON1 knockout mice are prone to atherosclerosis both induced by diet and apoE deficiency [156,157].
3. Over-expression of PON1 in mouse, rat, and rabbit models protects against atherosclerosis [135,158,159,160,161].

## 5. Objections to PON1 Being Essential for the Antioxidative, Antiglycative Role of HDL

There have, however, been reports critical of the proposal that a substantial component of the protection that HDL affords LDL against oxidative modification is due to its component PON1. These are as follows:

1. There are those that focus on the role of HDL in RCT in which so much research has been invested. They tend to dismiss the effect of HDL in decreasing LDL oxidation as due to the hydrolytic activity of PAFAH present on HDL [14,162,163,164]. Undoubtedly, HDL has PAFAH activity, and proteomic studies show PAFAH itself to be present in HDL. However, the majority of the PAFAH activity of HDL (not so LDL) is due to PON1. Furthermore, the effect of both whole HDL and partially purified PON1 in protecting LDL against oxidative modification is unaffected by inhibitors of PAFAH [39].
2. PON1 when highly purified loses its capacity to protect LDL against oxidation, therefore the effect of less highly purified PON1 is due to contamination by another enzyme truly responsible [165]. PON1 has hydrophobic domains and requires a lipid environment for its activity towards lipophilic substrates. It is difficult to maintain this lipid environment whilst achieving the final stage of purification, whether of PON1 from serum or of rPON1 from the culture media of genetically engineered cells [166,167,168,169,170].
3. There are numerous commercially available rPON1 preparations. Most, if not all, do not protect LDL against oxidation. Methods used to isolate the rPON1 have been optimized to maximize the yield with the aim of organophosphate hydrolysis. Initial screening is usually by phenyl acetate hydrolysis and then by testing potency as potential antidotes to organophosphate toxicity. The rPON1 thus produced may be lacking its hydrophobic environment depending on its method of isolation and may have structural modifications and tagging to enhance ease of isolation and potency as an organophosphate detoxicating enzyme. The antioxidant, antiatherogenic properties of PON1 were, however, reported to persist in rPON1 [171] prepared by one published method [172].
4. HDL can still protect LDL against oxidation in the absence of Ca^2+^ when paraoxon hydrolysis has thus been abolished [173]. However, soon after this report, it was revealed that Ca^2+^ is much less critical for phospholipid peroxide hydrolysis than for its aryl esterase and paraoxonase hydrolytic activities [174].
5. Mendelian randomization epidemiology based on the 192 polymorphism shows that the PON1 isoform is more active in promoting paraoxon hydrolysis. However, although this polymorphism is associated with ASCVD, in one report the association was not so strong as to rule out publication bias [175]. As has been earlier discussed, the impact of the R isoform on serum PON1 activity is not as strong as the authors of this report supposed. More recent meta-analyses have largely discounted publication bias as the explanation.
6. PON1 is the most active as a lactonase [149]. Although PON1 may have evolved as a lactonase, this does not mean that it is not involved in preventing a disease, such as atheroma, the clinical manifestations of which have only become apparent as a major epidemic in the last century [176]. If we allow that the protective effect of PON1 against ASCVD is not by protecting LDL against oxidative modification, then we must entertain that the discovery of its antiatherogenic role was serendipitous. Certainly, the possibility that its lactonase activity might be important, for example, against homocysteine thiolactone [177], should not be discounted, and further research should be directed in that area. However, the current evidence for an antioxidative role is compelling.

## 6. Conclusions

There is no doubt that HDL can protect LDL against oxidative modification. The extent to which HDL displays its antioxidative capacity is dependent on the balance between its pro- and antioxidant components, which varies between individuals, and on the propensity to develop certain diseases, such as ASCVD. Although primarily a lactonase, PON1 is the component of HDL that contributes most to its antioxidant activity. Epidemiology has revealed an inverse relationship between serum PON1 activity and ASCVD incidence. Experiments with genetically modified animals show convincingly that PON1 can prevent the development of atheroma. Furthermore, evidence suggests that in the circulation PON1 may protect LDL against oxidative modification, occurring, for example, as a consequence of hem metabolism, and against glycation due to glucose and its metabolites generated in the PPP and glycolysis pathway. OxLDL (and probably glycLDL) preferentially cross the arterial endothelium by receptor-mediated transcytosis, where their attempted removal by monocyte-macrophages creates foam cells. Within the arterial wall, additional oxLDL is generated by ROS secreted by inflammatory cells and leakage from cells generally when couplet oxygen is reduced. PON1 is important for the antioxidative mechanism by which HDL opposes atherogenesis, which may provide a better avenue of inquiry in the prevention and treatment of ASCVD [118] than has emerged from the emphasis on its role in RCT.
